# Decoding the Hayward kiwi (*Actinidia deliciosa* var Hayward) genome: transcriptomic responses to drought and salinity and *AdhSAP4’s* role in salinity stress responses

**DOI:** 10.3389/fpls.2025.1637092

**Published:** 2025-09-23

**Authors:** Samuel Parra, Gerardo Núñez-Lillo, Patricio Tapia-Reyes, Emerson Clovis Carrasco-Lozano, Vincenzo Porcile, Christian Gonzalez-Calquin, Leticia Amaza, Luis Felipe Quiroz, Claudio Meneses, Michael Handford, Lorena Norambuena, Juan Pablo Martínez, Claudia Stange

**Affiliations:** ^1^ Departamento de Biología, Facultad de Ciencias, Universidad de Chile, Santiago, Chile; ^2^ Escuela de Agronomía, Facultad de Ciencias Agronómicas y de los Alimentos, Pontificia Universidad Católica de Valparaíso, Quillota, Chile; ^3^ Escuela de Biotecnología, Facultad de Ciencias, Universidad Santo Tomás, Santiago, Chile; ^4^ Agriculture and Bioeconomy Research Centre, Ryan Institute, University of Galway, Galway, Ireland; ^5^ Departamento de Genética Molecular y Microbiología, Facultad de Ciencias Biológicas, Pontificia Universidad Católica de Chile, Santiago, Chile; ^6^ Departamento de Fruticultura y Enología, Facultad de Agronomía e Ingeniería Forestal, Pontificia Universidad Católica de Chile, Santiago, Chile; ^7^ Millennium Nucleus for the Development of Super Adaptable Plants (MN-SAP), Agencia Nacional de Investigación y Desarrollo (ANID)-Millennium Science Initiative Program, Santiago, Chile; ^8^ Millennium Institute Center for Genome Regulation (CRG), Santiago, Chile; ^9^ Instituto de Investigaciones Agropecuarias (INIA) Rayentue, Rengo, Chile

**Keywords:** Hayward kiwi (*Actinidia deliciosa*), salinity stress, drought stress, transcriptomic (RNA-seq), genome assembly, stress associated protein (SAP)

## Abstract

Abiotic stresses such as drought and salinity pose major limitations to crop productivity, particularly in sensitive species like the Hayward kiwi (*Actinidia deliciosa* var. Hayward). Stress-associated proteins (SAPs), defined by conserved A20/AN1 zinc finger domains, are emerging as key modulators of plant stress responses. Despite their relevance in model plants, their functional roles in kiwi remain unexplored. In this study we assembled a high-quality haploid reference genome for Hayward kiwi, annotating 42,797 protein-coding genes. RNA-Seq profiling of in vitro leaves exposed to drought (20% PEG-6000) and salinity (200 mM NaCl) at 6 and 24 hours identified differentially expressed genes (DEGs). The differential gene expression kinetics between drought and salinity suggest distinct adaptation mechanisms. Fourteen SAP genes were identified, with AdhSAP4 showing salt-induced expression and homology to rice OsSAP7. Nuclear localization of AdhSAP4-GFP was confirmed, and transgenic tobacco lines overexpressing AdhSAP4 exhibited heightened salt sensitivity, reduced growth, and chlorophyll loss. This study establishes a genomic and transcriptomic framework for kiwi stress biology and confirms AdhSAP4 as a negative regulator of salinity tolerance. The evolutionary conservation of SAP function highlights their potential as biotechnological targets for enhancing stress resilience in perennial crops like kiwi.

## 1 Introduction

Plants are sessile organisms that must adapt to environmental changes to survive. The increasing climatic variations that plants face in the current global warming scenario places great pressure on crops, which experience reduced yield and quality under these adverse conditions, putting food security at risk (Kuyper and Struik, 2014; Buck et al., 2023). Among the prevailing abiotic stress conditions that most adversely affect crop quality and yield are salinity and water scarcity (Farooqi et al., 2020; Ullah et al., 2021). At present, 62% of the cultivated land in the United States is affected by drought and 10% by salinity (Omuto et al., 2020).

Drought and salt stress dramatically reduce plant development and growth. Under moderate or severe drought, salt and ion accumulation in the soil causes osmotic stress and ion toxicity in plants. Increased drought stress decreases plant cell turgor pressure, making cell walls wrinkled and loose. This can reduce leaf size and number, fresh weight, and the water content, thus affecting most agronomically relevant crops (Fathi and Tari, 2016). Drought and salinity also affect nutrient absorption, causing osmotic and ionic stress that lead to oxidative stress, and reductions in photosynthesis and yield (Ullah et al., 2021). Plants have developed several cellular and molecular mechanisms to cope with these adverse environmental factors (Hualpa-Ramirez et al., 2024). At the cellular level, the high extracellular sodium (Na^+^) concentration disrupts cellular ion homeostasis, leading to an increase in cytosolic Na^+^. The most studied mechanisms in conferring tolerance to abiotic stress are the pathways dependent and independent of the phytohormone abscisic acid (ABA) (Zhu et al., 2002; Chen et al., 2020). ABA signal transduction cascades include the phosphorylation of ion channels and transcription factors, leading to physiological and molecular responses such as stomatal closure, production of osmoprotectants and synthesis of proteins involved in water retention (Cutler et al., 2010; Lim et al., 2015; Liu et al., 2022).

Positive abiotic stress regulators trigger the expression of genes required to cope with stress such as the transcription factors from the bZIP, NAC and MYTC/MYB families (Yang et al., 2025), as well as reorganizing the distribution of circulating proteins through the ubiquitination process. Additionally, negative regulators that are responsible for restoring the normal function of the cell are also produced regardless of whether the initial stress is still present either through gene regulation or proteasome protein turnover (Qin et al., 2008; You and Chan, 2015). ZmABI5 (ABA INSENSITIVE 5) is an example of a negative regulator, because it results in higher sensitivity to salinity (by increasing ionic toxicity (Na^+^ accumulation) and decreased K^+^ uptake), drought, and temperature when overexpressed in *Nicotiana tabacum* (Yan et al., 2012). On the other hand, *ZmABI5* knockout mutants showed improved salt tolerance, reduced oxidative damage, and better ion homeostasis compared to wild-type plants (Yan et al., 2012).

In recent years, several studies on plant stress responses have highlighted the novel Stress Associated Proteins (SAPs), which have been shown to participate in a wide range of abiotic stress responses as positive or negative regulators (Qadir and Shams, 1997; Lloret et al., 2017; Bae et al., 2021; Wang et al., 2021). The SAP family members harbor the highly conserved A20/AN1-type Zinc finger domains (Giri et al., 2013; Vij and Tyagi, 2006). Zinc finger proteins are an important superfamily in eukaryotes that have been commonly found to be involved in responses to environmental stresses. The A20 domain contains a conserved CX_2-4_CX_11_CX_2_C domain and the AN1 domain was discovered as a ubiquitin-like fusion protein (Giri et al., 2013; Linnen et al., 1993). In the genomes of *Arabidopsis thaliana* and *Oryza sativa*, 14 and 18 SAP-related proteins have been identified, respectively (Vij and Tyagi, 2006). The evidence accumulated over the last decade indicates that SAP proteins participate in the proteasome mediated rapid turnover of proteins, possibly acting as E3 ubiquitin-ligases in the ubiquitin-proteasome system (UPS) which are translocated to the nucleus to bind cis-acting elements of stress responsive genes (Vij and Tyagi, 2008; Giri et al., 2013). Specifically, *OsiSAP7* confers a lower survival rate under drought in transgenic rice (Sharma et al., 2015) which suggests that it codes for a negative regulator of drought tolerance in this species.

Kiwi (*Actinidia deliciosa*) is a commercially valuable species worldwide. The Hayward variety, originally from New Zealand, has remained the most cultivated and commercialized variety in recent years (Ferguson et al., 2023). It grows very well in well-drained soils, but is very sensitive to drought, salinity and environmental changes, conditions that seriously affect fruit production, yield and quality (Maghradze et al., 2011; Zhong et al., 2019; Abid et al., 2020; Quiroz-Iturra et al., 2022).

With the rapid advancement of omics technologies, there is an unprecedented opportunity to unravel the specific molecular responses of the Hayward variety of kiwifruit to acute drought and saline stresses. This study aims to investigate the genome-wide transcriptional dynamics triggered by these abiotic challenges, providing insight into the initial stress-adaptive mechanisms of a commercially relevant cultivar. By focusing on key regulatory genes and their molecular features, we seek to identify potential targets for genetic improvement and develop a deeper understanding of the mechanisms governing stress tolerance in perennial fruit crops. The findings are expected to contribute to sustainable agricultural practices and resilience strategies in the face of climate-induced environmental stresses.

## 2 Materials and methods

### 2.1 Plant material

*Nicotiana tabacum* (cultivar Xanthi NN) and T1 *p35S:AdhSAP4-GFP* transgenic lines were cultivated in the greenhouse in glass wool irrigated with a standard hydroponic medium (0.125 mM KNO_3_, 0.15 mM Ca(NO_3_)_2_·4H_2_O, 0.075 mM MgSO_4_·7H_2_O, 0.05 mM KH_2_PO_4_, 5 µM KCl, 5 µM H_3_BO_3_, 1 µM MnSO_4_, 200 nM ZnSO_4_·7H_2_O, 150 nM CuSO_4_, 10 µM Na_2_O_3_Si, 10 µM Fe/DTPA, pH 6). Kiwi plants (*Actinidia deliciosa* cv. Hayward) were a gift of Viverosur (https://viverosur.com/, accessed on 12 January 2024). Internodal explants of kiwi plants were surface sterilized using 70% ethanol (30 s) and incubated in 10% (v/v) sodium hypochlorite and 1 drop of Tween 20 (20 min). For *in vitro* plant regeneration, kiwi explants were then washed four times with sterile water and placed in flasks in solid half strength MS medium (2.2 g/L MS salts, 3% sucrose, 0.22% vitamins, 0.01% myo-inositol and 0.7% agar pH 5.7), supplemented with 0.5 mg/L BAP. Explants were subcultured monthly until shoot regeneration (Quiroz-Iturra et al., 2022). Plants were maintained at 24°C under a 16 hrs. photoperiod illuminated with white, fluorescent light (150 µmol m^−2^ s^−1^ at 22 – 25°C).

### 2.2 Drought and salinity treatment

Two-month-old *in vitro* unrooted kiwi plants were grown in solid MS medium containing 0.44% MS salts, 2% sucrose, 0.01% myo-inositol, 0.7% agar, pH 5.8 supplemented with 200 mM NaCl (salt treatment). For transcriptomic analysis, leaves from three plants were taken and pooled for RNA extraction, three replicate samples were taken at the beginning of the assay (CT0), at 6 hrs. (ST1) and at 24 hrs. (ST2). For drought treatment, unrooted shoots were placed in liquid MS medium supplemented with 20% PEG-6000 submerging only the stem. Samples were taken at CT0, at 6 hrs. (DT1) and at 24 hrs. (DT2).

For chronic tobacco salinity assays, 3-month-old six wildtype and six T1 *p35S:AdhSAP4-GFP* plants of four different lines were irrigated for one month twice per week with MS supplemented with or without 200 mM NaCl. At harvesting, the third and fourth leaves were used to determine fresh weight, dry weight and chlorophyll using a SPAD-502 chlorophyll meter (Konica Minolta). For seedling tobacco salinity survival experiments, nine wildtype (WT) and T1 *p35S:AdhSAP4-GFP* seeds were surface sterilized and grown on solid MS with vitamins + 1% sucrose for 21 days. Then, nine seedlings of each genotype were sown in solid MS with vitamins + 1% sucrose. For salt stress treatments, 100, 150 and 200 mM NaCl was added. Plant growth were measured three times every week and analyzed using the GRID Python package with default settings for K-Means clustering algorithm (K = 3) and channel binarization from Zhiwu Zhang laboratory https://zzlab.net/GridFree (Hu and Zhang, 2021). Statistical analysis was performed using one-way ANOVA with a Tukey-HDS test with a *p-value* of 0.05 in a custom Python script using Statsmodels library.

### 2.3 DNA extraction

DNA was obtained from *N. tabacum* and *A. deliciosa* leaves according to Doyle (1991) with slight modifications (Quiroz-Iturra et al., 2022). One-hundred mg of fresh leaves were ground using liquid nitrogen to a fine powder, then 2 mL CTAB buffer at 60°C (2% CTAB, 2 M NaCl, 20 mM EDTA, 1% PVP40, 100 mM Tris) and 5 µL β-mercaptoethanol were added and homogenized. The mixture was incubated for 15 min at 60°C with regular mixing. After a 5 min incubation, one volume of ice-cold chloroform:isoamyl alcohol (24:1) was added. The samples were mixed and centrifuged at 10,000 g for 10 min. The aqueous upper layer was transferred to a new tube containing one volume of ice-cold isopropanol and precipitated for 30 min at -20°C. Then, samples were centrifuged at 10,000 g for 10 min and the pellet was washed with 70% ethanol and centrifugated at 10,000 g for 5 min. The pellet was dried and resuspended in 50 µL of nuclease free water. The suspension was mixed with one volume of a phenol:chloroform:isoamyl alcohol (25:24:1) and centrifugated at 10,000 g for 5 min. The upper aqueous phase was transferred to a new tube containing one volume of chloroform:isoamyl alcohol (24:1) and mixed. After a 10,000 g centrifugation for 10 min, the aqueous upper phase was transferred to a tube containing one volume of ice-cold 70% ethanol. After a further 10 min centrifugation at 10,000 g, the pellet was dried using a speedvac and resuspended in 50 µL of nuclease free water.

### 2.4 Illumina genome sequencing

The paired-end sequencing library was prepared using an Illumina, Truseq Nano DNA LT Library Preparation Kit (Illumina, California, United States). 100 ng of gDNA was sheared using a fragmentase (Bioruptor Pico, Diagenode) to generate a mean fragment distribution of 320 bp. The fragments were then subjected to end repair using end repair mix and indexing adapters were ligated to the ends of the DNA fragments. The ligated products were purified using SP beads supplied in the kit. The size-selected product was PCR amplified as described in the kit protocol. The amplified library was analyzed in a Bioanalyzer 2100 (Agilent Technologies, California, USA) using a DNA 1000 chip (Agilent Technologies) as per the manufacturer’s instructions. The library was then loaded onto the Illumina HiSeq platform for cluster generation and subjected to paired-end sequencing.

### 2.5 PacBio whole genome sequencing

Genomic DNA libraries were prepared using the SMRTbell prep kit 3.0 according to the manufacturer’s procedure (PacBio). DNA was fragmented with g-TUBE to the recommended length of 15–18 kb fragments and cleaned with SMRTbell cleanup beads. Fragmented DNA was processed for nick repairing, A-tailing and end repairing. The SMRTbell adapter was ligated to the prepared DNA fragments and then the ligation products were treated with exonuclease to degrade failed ones. Finally, fragments with the desired size were selected by BluePippin and were considered ready for sequencing in PacBio Sequel II (Pacific Biosciences, California, USA).

### 2.6 RNA extraction

RNA extraction was performed following the protocol described by Chang et al. (1993) with modifications (Parra, 2023). Two-hundred mg of fresh *in vitro*-grown kiwi leaves were ground with liquid nitrogen to a fine powder and resuspended in 3 mL extraction buffer containing 2% CTAB, 2% PVP, 100 mM Tris-HCl (pH 8.0), 25 mM EDTA, 2.0 M NaCl and 0.5 g/L spermidine. β-mercaptoethanol (2%) was added just before use. After incubating the mixture at 65°C, an equal volume of chloroform:isoamyl alcohol was added and centrifuged for 10 min at 13,000 rpm at room temperature. The aqueous phase was precipitated overnight at 4°C adding ¼ volume of 10 M LiCl. Precipitated RNA was sedimented by centrifugation at 13,000 rpm at room temperature. The pellet was dissolved with a volume of buffer containing 1 M NaCl, 0.5% SDS, 10 mM Tris-HCl (pH 8.0), and 1 mM EDTA (pH 8.0), and then mixed by vortexing with an equal volume of chloroform:isoamyl alcohol and centrifugated as before. The aqueous phase was precipitated with 2 volumes of ice-cold ethanol for 2 hrs. at -20°C. Finally, the precipitated RNA was recovered by centrifugation for 10 min at 13,000 rpm at 4°C and then resuspended in 30 µL of nuclease free TE buffer.

### 2.7 RNA library construction and sequencing

RNA libraries were prepared with the TruSeq Stranded mRNA sample preparation kit (Illumina) according to the manufacturer’s protocol preserving strand specificity, using 300 ng of total RNA from each sample. The polyadenylated RNA was purified with oligo-dT magnetic beads, and the poly(A) RNA was fragmented with divalent cations under elevated temperature. First-strand cDNA synthesis produced single-stranded DNA copies from the fragmented RNA by reverse transcription. After second-strand cDNA synthesis, the double-stranded DNA underwent end repair, and the 3′ ends were adenylated. Finally, universal Illumina adapters were ligated to the cDNA fragments, and PCR was performed to produce the final sequencing library. After validation of the library using a DNA 1000 chip (using an Agilent Technologies 2100 Bioanalyzer), the samples were pooled together in equal concentrations and run on an Illumina HiSeq for 150 cycles of paired-end sequencing. Each RNA-seq sample yielded between 39 and 59 million paired-end reads, with an average sequencing depth of approximately 47 million reads per sample (**Supplementary Table S7**). RNA library construction and sequencing processes were performed by the Genoma Mayor Sequencing service (Universidad Mayor, Santiago, Chile).

### 2.8 *A. deliciosa* cv. Hayward reference genome assembly and annotation

Adapter-free Illumina and PacBio libraries were assembled with MaSuRCA v4.0.9 software (Zimin et al., 2017) using the FLYE assembler and a “jf_size” of 2,100,000,000. The obtained contigs were filtered with Purge_Dups v12.5 software (available at https://github.com/dfguan/purge_dups) and then rearranged as scaffolds using the *A. chinensis* var. Hong Yang v3.0 genome as target (Wu et al., 2019) with RagTag v2.1.0 software (Alonge et al., 2022). Genome repetitive elements were detected *de novo* with RepeatModeler v2.0.4 (Flynn et al., 2020) and then masked from the genome with Repeatmasker v.4.1.5 (Tarailo-Graovac and Chen, 2009). For gene prediction, MAKER v3.01.3 (Campbell et al., 2014) software was used, which requires transcript and protein evidence to generate gene prediction. Filtered adapter-free RNA-seq reads from this and previous studies (NCBI Bio-Project PRJNA564374), were used as transcriptomic evidence in addition to the protein sequences available from *Viridiplantae* in Uniprot/Swiss-Prot (www.uniprot.org). Predicted genes were annotated using InterproScan v5.62-94.0 (Jones et al., 2014) and eggNOG-mapper v2.1.9 (Cantalapiedra et al., 2021), against the eggNOG v5.0 database (Huerta-Cepas et al., 2019). Genome assembly and annotation completeness was evaluated with BUSCO v.5.4.7 software (Seppey et al., 2019) against the embryophyta_Odb10 dataset.

### 2.9 Identification of orthologous groups

For comparative genomics analysis, annotated proteins were extracted from each of the following genomes: *A. chinensis* Russel, *A. chinensis* Red5 (Pilkington et al., 2018), *A. chinensis* Hong yang (Wu et al., 2019), *A. eriantha* (Tang et al., 2019) and *Vaccinum darrowii* (Yu et al., 2021) as an outgroup. These proteomes were compared using the OrthoVenn3 platform in its web version (https://orthovenn3.bioinfotoolkits.net). OrthoFinder (Emms and Kelly, 2019) with an e-value of 1e^-3^ and an inflation value of 1.5 for the comparison of orthologous groups. The ultrametric phylogenetic tree was generated using the maximum likelihood algorithm with the LG+CAT evolutionary model obtained from FastTree2 software (Kumar et al., 2022). The calibration of the tree was performed using the distance in millions of years between *A. eriantha – A. chinensis* Hongyang (12.3 MYA) and *A. errianta* – *V. darrowii* (91 MYA). Both distances were obtained from http://www.timetree.org/. For the calculation of orthologous gene family expansions and contractions, the CAFE5 software (Mendes et al., 2020) was used with a lambda value of 0.005906147620034 and maintaining the rest of the default settings.

### 2.10 Phylogenetic tree of SAP homologous genes

Protein sequences annotated as SAPs were extracted from the *A. thaliana* and *O. sativa* genomes. Amino acid sequences were obtained from the annotated genomes of *A. chinensis* Russel, *A. chinensis* Red5, *A. chinensis* Hong yang and *A. deliciosa* Hayward (this article) for each of the genes belonging to ortholog clusters 560, 6382, 6815, 8272, 8957, 11951, 12275, 15133 and 16792 (**Supplementary Table S1**).

From this collection of 93 sequences, a multiple alignment was generated using Mafft v7.511 software with the options “–averagelinkage –reorder –anysymbol –maxiterate 2 –retree 1 –globalpair” and the result was transformed to CLUSTAL format and a maximum-parsimony phylogenetic tree was generated using IQ-TREE v1.6.12 (Nguyen et al., 2015) with the options “st AA -m TEST -bb 1000 -alrt 1000”. The model selected by the software was WAG+G4. A 1000 bootstrap analysis was then performed (Hoang et al., 2018) from which the consensus tree was generated. The consensus tree was modeled using the online software iTOL (Letunic and Bork, 2024). For the SAP phylogenetic tree from the genome of *A. deliciosa* var. Hayward, a multiple alignment was generated using the software Mafft v7.511 software with the options “–averagelinkage –reorder –anysymbol –maxiterate 2 –retree 1 –globalpair” and the result was transformed to CLUSTAL format and a maximum-parsimony phylogenetic tree was generated using IQ-TREE v1.6.12 with the options “st AA -m TEST -bb 1000 -alrt 1000”; the model selected by the software was WAG+G4. A 1000 bootstrap analysis was then performed from which the consensus tree was generated. The consensus tree was modeled using the online software iTOL.

For the identification of protein domains, the NCBI web CD-Search tool (Wang et al., 2023) was used against the CDD database with an Expect value threshold of 0.01. These identified domains were manually plotted using the Mydomains:image Creator web application of the ProSite platform (Sigrist et al., 2012).

### 2.11 Differential expression analysis

Adapter-free sequenced reads were aligned with STAR v2.7.10 software (Dobin et al., 2013) against the *A. deliciosa* var. Hayward reference genome described in section 2.2.1. For each aligned library, the Bioconductor-Rsubread package v2.8.1 (Liao et al., 2019) was applied to assign expression values to each uniquely aligned fragment. Differential gene expression analysis was performed using the R package edgeR v3.36.0 (Robinson et al., 2010) using a trimmed mean of M-values (TMM) normalization method. To evaluate the obtained expression data, a heatmap and a principal component analysis (PCA) were performed with all normalized gene values using the pheatmap v1.0.12 software and the ggfortify R package v0.4.14 (Tang et al., 2016), respectively. Due to the wide dispersion between control replicates, it was decided to eliminate the CT0_2 sample from the differential expression analysis. DEGs were selected with an FDR < 0.01 and a |log_2_FC| > 2.0 by comparing each treatment against control samples and then plotting in a volcano plot using the R package EnhancedVolcano v1.14.0 (Blighe et al., 2022). The DEG lists were compared in a Venn diagram constructed using the Venny v2.1.0 software available online at https://bioinfogp.cnb.csic.es/tools/venny/. To search for genetic processes and pathways overrepresented in the DEG lists, a genetic enrichment analysis was performed using the Genetic Ontology (GO) database with the R package ClusterProfiler v4.0.5 (Yu et al., 2012) using *A. thaliana* DEG orthologs.

### 2.12 RT-qPCR for candidate gene validations

To validate transcriptome analysis, the same RNA samples used in the RNA-Seq analysis was used to synthesize cDNA. One μg of RNA was treated with DNAseI, RNAse-free enzyme (Thermo scientific) according to the manufacturer’s instructions. RNA samples were used to synthesize cDNA using the ImProm-II ™ Reverse Transcriptase (Promega) kit following the manufacturer’s instructions. Real time RT-PCR experiments were performed in an Agilent Stratagene Mx3000p machine, using Biotium Forget-me-not™ EvaGreen^R^ qPCR Master mix, as described in Quiroz-Iturra et al. (2022). Specific primers targeting the selected genes from the differential gene expression analysis were designed (**Supplementary Table S2**) using the Primer3 tool of Geneious Prime v2024.03 software. Gene expression levels were determined using the 2^−△△^Ct method (Livak and Schmittgen, 2001). Each RT-qPCR reaction was performed using three biological replicates and each sample was analyzed twice (technical replicate). In all cases, the reaction specificities were tested with melting gradient dissociation curves and electrophoresis gels. To test for significant differences in gene expression, two tailed Student t-tests (confidence interval 95%) were performed using the General Linear Models option in the statistical software package Graphpad Prism.

### 2.13 Vector construction

The *AdhSAP4* cDNA (504 bp) was obtained by RT-PCR from leaves using primers listed in **Supplementary Table S2** employing the Q5^R^ High-Fidelity DNA polymerase enzyme (New England Technologies). The amplified fragment was adenylated, purified and cloned into the pCR^®^8/GW/TOPO vector (Invitrogen) following the manufacturer’s instructions. Positive clones selected by enzymatic digestion with AvaII (Thermo scientific) were sequenced by Macrogen Corp. (USA). A positive pCR8/*AdhSAP4* clone was recombined into the pGWB5 vector using the Gateway™ LR-clonase II enzyme mix (Thermofisher) according to the manufacturer’s protocol generating the p*35S:AdhSAP4-GFP* binary vector. Positive clones were selected by enzymatic digestion with EcoRI (**Supplementary Figure S1**) and then introduced into *Agrobacterium tumefaciens* (GV3101 strain) for stable *AdhSAP4-GFP* expression in tobacco (**Supplementary Figure S2**). CDS integrity was determined by sequencing the *AdhSAP4* sequence and aligning the result with the *A. chinensis* Red5 reference (**Supplementary Figure S3**). Positive clones were grown in 5 mL LB liquid medium supplemented with Rifampicin (10 mg/L), Gentamicin (50 mg/L) and Kanamycin (100 mg/L) at 28°C with constant shaking for 12 hrs. Then, 200 µL of pre-culture was used to inoculate 30 mL LB liquid medium supplemented with the antibiotics Rifampicin (10 mg/L), Gentamicin (50 mg/L) and Kanamycin (100 mg/L). The culture was grown at 28°C with constant shaking for 12 hrs. or until an OD600 of 0.4-0.8 was reached. Then, the culture was centrifuged at 4,000 g for 10 min. The pellet was resuspended in liquid MS medium (0.44% MS medium with vitamins, 2% sucrose, 0.01% myo-inositol, pH: 5.8) supplemented with 200 μM acetosyringone.

### 2.14 Subcellular localization of AdhSAP4-GFP

To determine the subcellular localization of AdhSAP4, the *p35S:AdhSAP4:GFP* binary vector was transiently expressed in 2-month-old tobacco leaves by agroinfiltration (Flores-Ortiz et al., 2020). Samples were visualized in a Zeiss LSM5 confocal microscope at 498–525 nm (GFP) and 640–720 nm (Chlorophyll) 3 days after infiltration and images were processed with the ImageJ software.

### 2.15 Agrobacterium-mediated *Nicotiana tabacum* stable transformation

*In vitro* tobacco plants were cultivated for 6 weeks in solidified Murashige and Skoog (MS; 4.4 g/L MS salts, 20 g/L sucrose and 0.7% agar) medium and transformed according to Acevedo et al. (2023). Explants from petioles and leaves (~1 cm) were submerged and kept in suspension with *A. tumefaciens* carrying *p35S:AdhSAP4-GFP*. After 20 min, explants were placed on plates with solid half strength MS medium (2.2 g/L MS salts, 3% sucrose, 0.22% vitamins, 0.01% myo-inositol and 0.7% agar pH 5.7) in the dark at 24 °C for 48 hrs. Afterwards, explants were rinsed with sterile water, dried and maintained in MS medium supplemented with 50 mg/L kanamycin, 0.5 mg/L 6-BAP and 1 mg/L Indole-3-Butyric Acid for somatic organogenesis (Rosas-Saavedra et al., 2023). After shoots appeared (week 8-12), they were moved to half strength MS for elongation and greenhouse acclimatation (**Supplementary Figure S4**).

## 3 Results

### 3.1 *A. deliciosa* var. Hayward reference genome

From the genomic data sequenced by Pacbio and Illumina, a haploid genome of 567.6 Mbp was assembled with a BUSCO completeness of 96.2% (**Table 1**, **Supplementary Table S3**). 44.2% of the genome is composed of repetitive elements, the predominant ones being LTR Copia and Ty3-retrotransposons type retro-transposons accounting for 8.91% and 8.62% of the genome correspondingly, followed by Hobo-Activator DNA transposons with 3.92% of the genome (**Supplementary Tables S4, S5**). Using the Maker annotation pipeline, 42,797 protein-coding genes were identified (**Table 1**) all with an AED < 0.75, of which 94.75% were assigned some function based on homology with other proteins (**Supplementary Table S6**).

**Table 1:** *Actinidia* sp. genome comparison.

Reference name	Hayward	HongYang	Red5	Russell
*Actinidia* species	deliciosa	chinensis	chinensis	chinensis
Genome version	v1	v3	v2	v2
Genome size (Mbp)	567.6	583.2	595.9	618.6
Ploidy (2n)	6x=174	2x=58	2x=58	2x=58
Protein-coding genes	42,797	40,466	32,635	33,833
Genome covered with genes* (%)	44.61	37.09	30.38	32.77
Genome covered with repeats (%)	44.20	43.42	–	41.47
CG (%)	35.39	35.57	35.45	35.43
Completed BUSCOs* (%)	96.20	96.30	98.70	97.50
Unique BUSCOs (%)	73.20	80.00	70.30	66.50
Duplicated BUSCOs (%)	23.00	16.30	28.40	31.00
Fragmented BUSCOs (%)	1.40	1.20	0.40	0.60
Missing BUSCOs (%)	2.40	2.50	0.90	1.90

*1614 Total BUSCO genes embryophyta_db10.

*Gene covered includes coding sequences (CDS) and non-coding regions within gene models, introns and untranslated regions.

**Figure 1:**
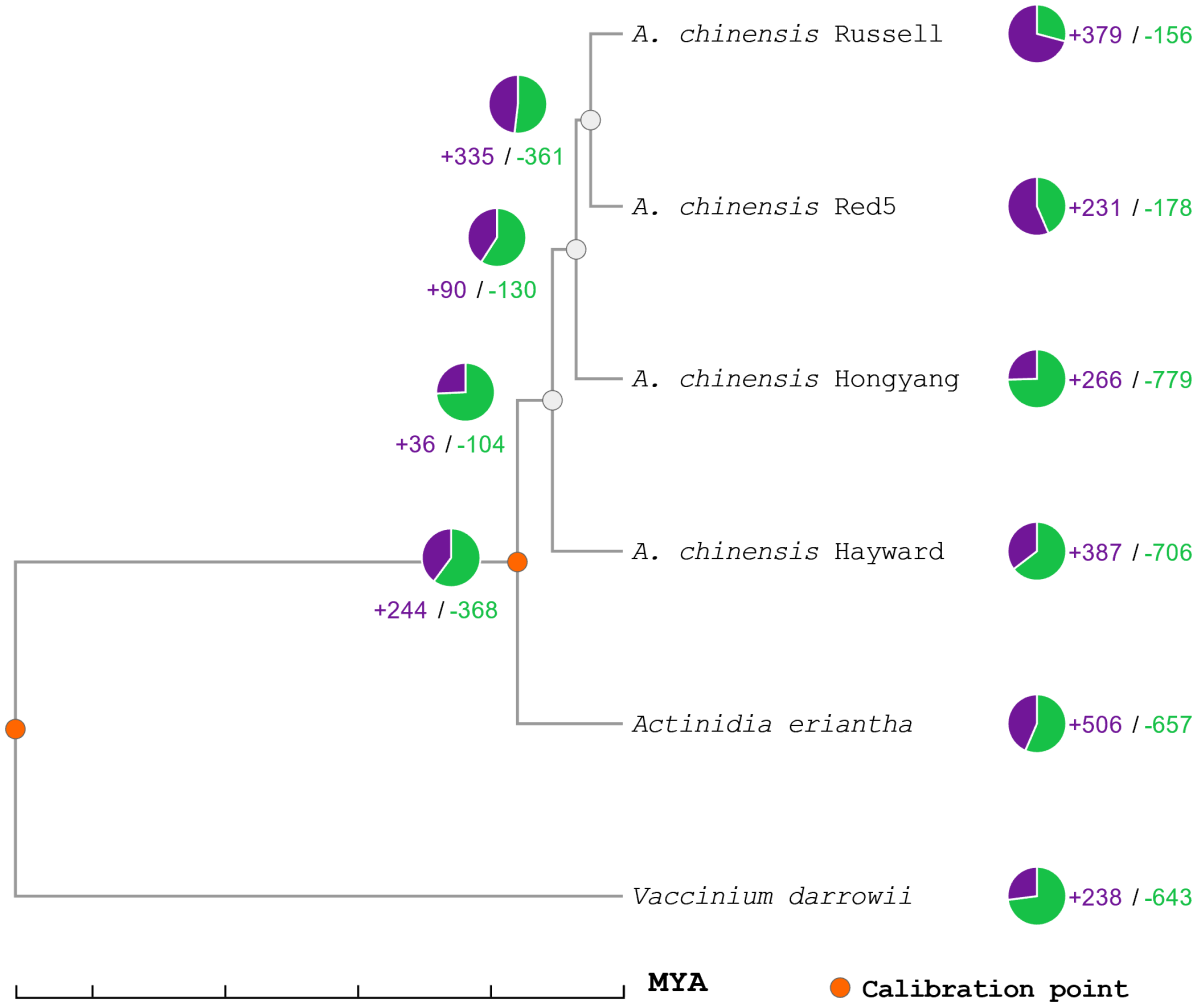
Phylogenetic tree of expansion and contraction of orthologous gene families in Actinidia. Orange circles represent calibration points for genetic distance, green numbers and graphs represent contractions of orthologous gene families, while purple numbers and plots represent expansions of orthologous gene families. MYA is millions of years ago. From the orthologous clusters, seven families of proteins associated with SAP genes were identified, with a total of 56 genes, of which 40 correspond to genes of the varieties of *Actinidia chinensis* and *Actinidia deliciosa* var Hayward (ST6).

The predicted proteins from the genome of *A. deliciosa* var. Hayward were used with those of *A. chinensis* var. Russel, *A. chinensis* var. Red5, *A. chinensis* var. Hongyang, *A. eriantha* and *V. darrowii* in the search for orthologous group expansions and contractions (**Figure 1**). As can be seen, the divergence of *A. deliciosa* var. Hayward from the *A. chinensis* clade is less than 11 MYA, with the expansion of at least 387 orthologous gene clusters compared to their most recent common ancestor, and a contraction of 706 orthologous gene clusters.

### 3.2 Transcriptomic analysis of kiwi leaves in drought and salinity stress treatments

#### 3.2.1 RNA sequencing and differential gene expression analysis

**Figure 2:**
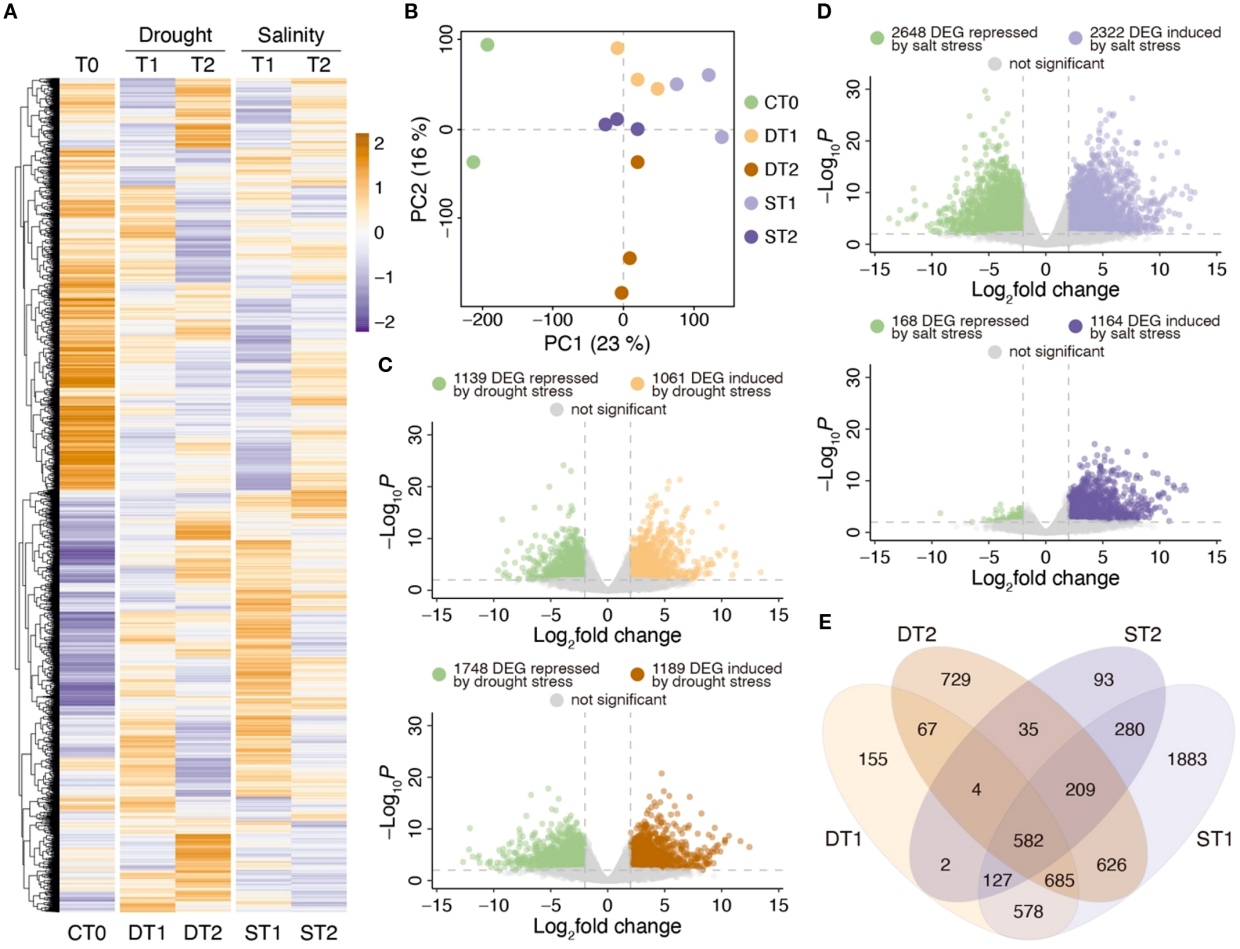
Transcriptomic analysis of kiwi leaves exposed to drought and salinity stress treatments. **(A)** Purple-orange color scale heatmap considering all differential expressed genes (DEGs) in at least one condition with an FDR < 0.01. All expression data was scaled considering the average of each gene divided by SD. CT0: Control without treatment, DT1: Drought 6 hrs, DT2: Drought 24 hrs, ST1: Salt 6 hrs, ST2: Salt 24 hrs of treatment. Each column represents the replicate average expression value of each condition analyzed. **(B)** Principal component analysis using all expression data. **(C)** Volcano plots representing the number of DEGs associated with drought stress between DT1 *vs* CT0 (upper panel) and DT2 *vs* CT0 (lower panel). **(D)** Volcano plots representing the number of DEGs associated with salinity stress treatments between ST1 *vs* CT0 (upper panel) and ST2 *vs* CT0 (lower panel). **(E)** Venn diagram comparing the number of DEGs in drought (DT1 and DT2) and salinity (ST1 and ST2) treatments.

The results obtained for RNA extraction for sequencing are detailed in **Supplementary Table S7**. After sequencing, an average of 46,945,521 reads were obtained with approximately 93.6% of the reads with QC > 30.0. Approximately 89.7% of total reads were correctly aligned against the *A. deliciosa* var. Hayward reference genome, which is equivalent to an average of 42,110,341 reads per library. Four candidate genes (*AdhNCED, AdhNAC, AdhPUB24* and *AdhSOS*) obtained from transcriptomic analysis were used to validate RNA-seq expression data (**Supplementary Figure S5**). The differential expression analysis was performed by comparing leaf samples from plants exposed to stress (drought and salinity) for 6 hrs. (T1) and 24 hrs. (T2) of treatment against control samples without stress. In total, 10,459 DEGs were identified in any of the stress treatments with an FDR < 0.01. The normalized and scaled expression of these DEGs was represented in a heatmap (**Figure 2A**), identifying significant differences between the responses to drought and salinity stress and at T1 and T2. Replicate distribution is shown in the PCA of **Figure 2B**. With 23% variability, component 1 shows the separation between control samples and samples with some stress treatment, while component 2 separates samples at 6 hrs. (T1) and 24 hrs. (T2) of the drought treatment with 16% of variability.

The differential expression analysis between control and drought stress samples is shown in **Figure 2C**. Using a |log_2_FC| > 2.0, a total of 2,200 DEGs were identified in response to 6 hrs. of drought stress, while after 24 hrs. of treatment, the stress response was increased, identifying a total of 2,937 DEGs. It should be noted that from 6 to 24 hrs. of treatment, considerable increases in the number of induced genes (from 1,061 to 1,189 DEGs) and repressed genes (from 1,139 to 1,748 DEGs) were also observed. On the other hand, when comparing control samples to those with 6 hrs. of salt stress (**Figure 2D**), 4,970 DEGs were identified (2,648 repressed and 2,322 induced by salinity), while after 24 hrs. of stress, 1,332 DEG were found, of which only 168 DEG were repressed and 1,164 were induced by salinity.

The Venn diagram in **Figure 2E** shows a group of 582 common DEGs for both stress treatments (drought and salinity) at both treatments (T1 and T2). Interestingly, in drought stress, there are more unique drought stress DEGs at 24 hrs. (DT2; 729 DEG) than at 6 hrs. (DT1; 155 DEG), while in salinity stress treatments, the greatest number of unique DEGs was observed at 6 hrs. of treatment (ST1; 1883 DEG), a response which decreased drastically at 24 hrs. (ST2; 93 DEG). All these results suggest that in kiwi leaves, there is a faster response to salt stress than to drought stress treatments, and that after 24 hrs. of salt stress the plants begin to return to normal conditions when compared to control plants (CT0).

#### 3.2.2 Gene ontology analysis

**Figure 3:**
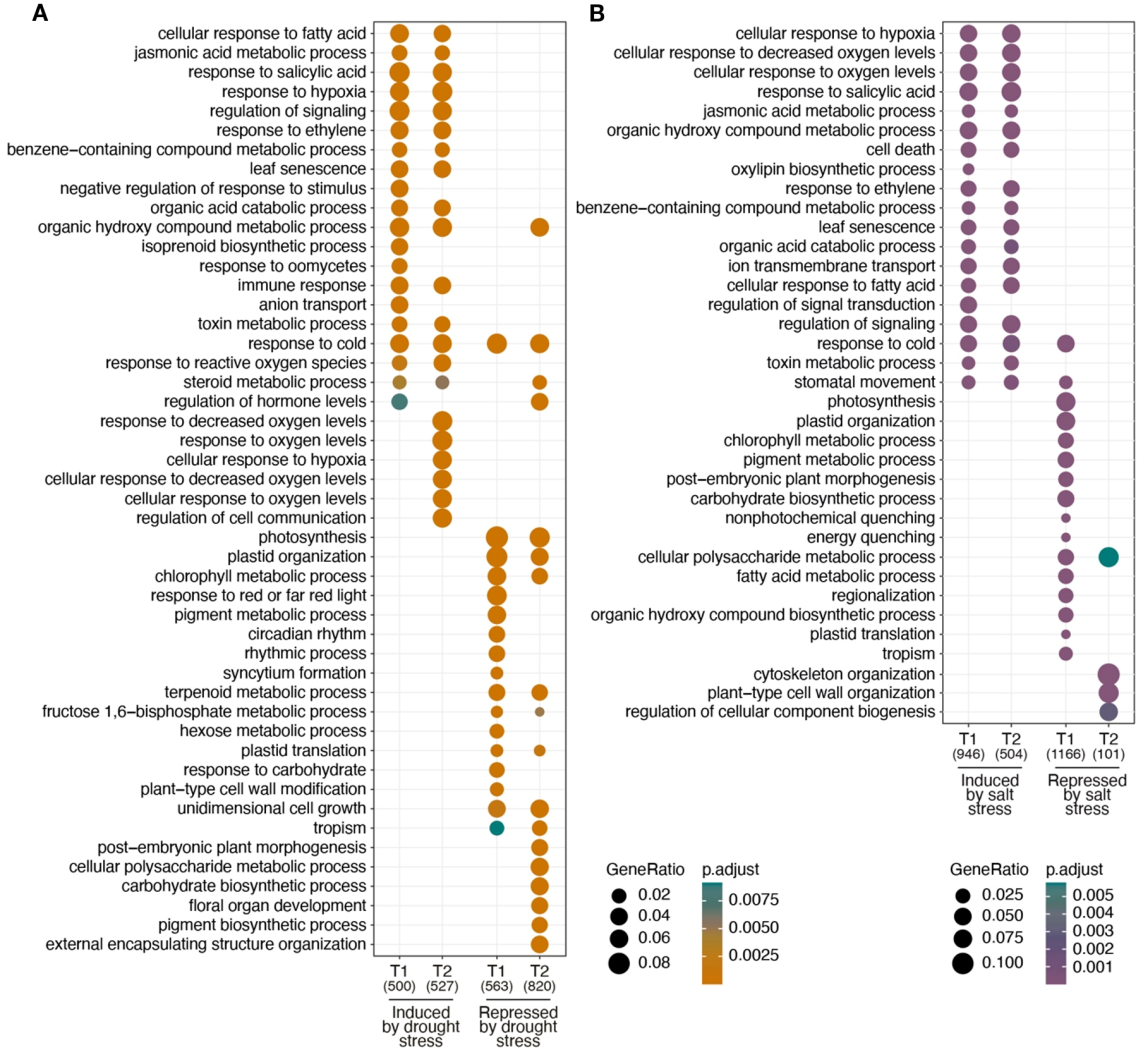
Comparative gene ontology analysis. GO analysis using the identified DEGs between control samples (CT0) and drought stress (DT1 and DT2) treatments **(A)** and for salinity stress (ST1 and ST2) treatments **(B)**.

To determine the overrepresentation of gene functions in each of the DEG groups identified in drought and salinity stress treatments, a comparative GO analysis was carried out between the different stress treatments (**Figure 3**). Regarding the upregulated DEGs, kiwi presents a similar response to both types of stress treatments revealing that phytohormones may play a key regulatory role. Jasmonic acid, organic hydroxy compounds and toxin metabolic processes, responses to salicylic acid, ethylene, fatty acids and cold, leaf senescence, regulation of signaling and organic acid catabolic processes are overrepresented in both drought and salt stress treatments at T1 and T2 (**Figures 3A, B**). However, notable differences emerged in the response to hypoxia and decreased oxygen levels in the GO terms. Under drought stress (**Figure 3A**), these GO terms were identified late in T2, whereas in salt stress (**Figure 3B**), they were strongly represented at both treatment times, T1 and T2. On the other hand, regarding the GO terms overrepresented in the downregulated DEG groups, photosynthesis, plastid organization and chlorophyll metabolic processes stood out in drought stress treatments at T1 and T2, but in salinity stress they were only represented at T1. Other GO terms such as post-embryonic plant morphogenesis, cellular polysaccharide metabolic processes and the carbohydrate biosynthetic processes were represented at T1 in salinity stress treatments and at T2 in drought stress treatments. These results suggest that there is a temporally differential response to drought and salt stress treatments, supporting previous results (**Figure 2D**) showing that plants after 24 hrs. of salt stress begin to return to prestress conditions.

#### 3.2.3 Identification of drought/salinity-stress response regulatory candidate genes

**Figure 4:**
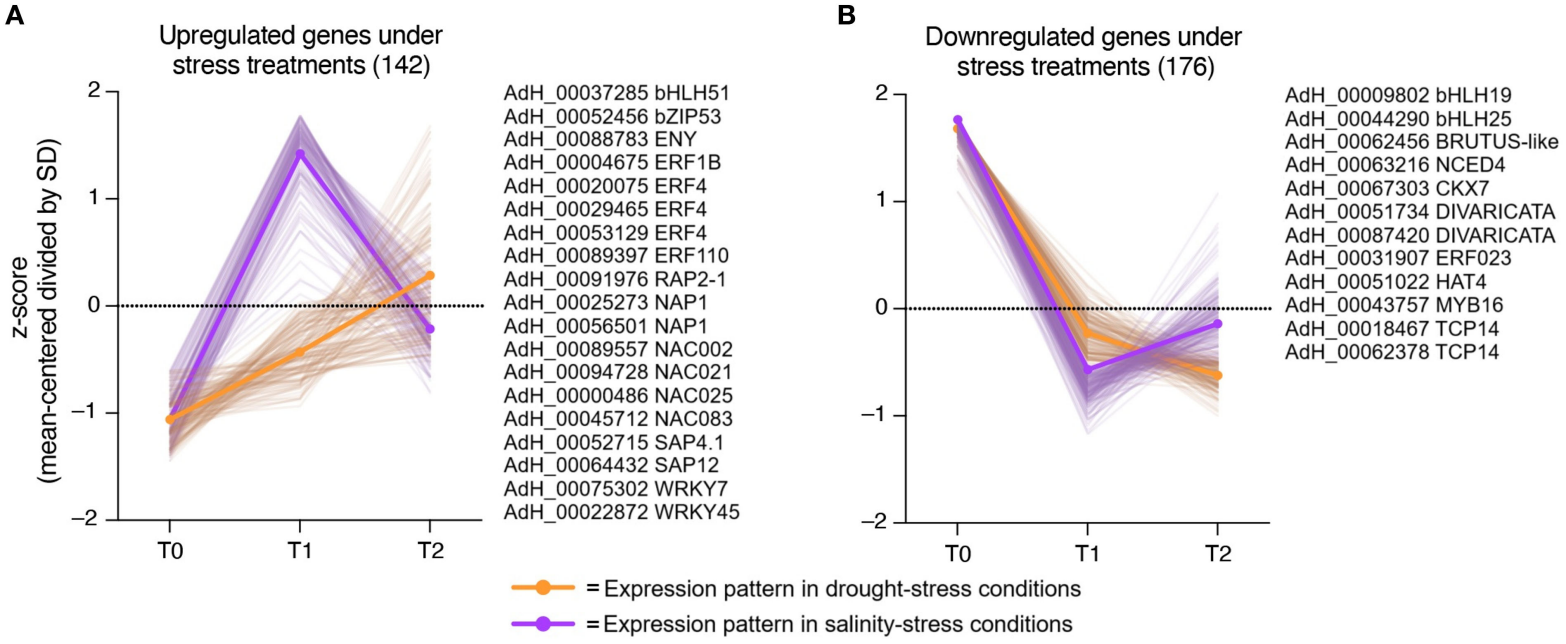
Expression pattern under drought and salinity and selection of stress-related regulatory candidate genes. Expression patterns of genes highly correlated with the plant drought- and salinity-stress responses were represented by orange and purple continuous lines, respectively. Expression values were scaled considering the mean centered divided by SD (z-score). **(A)** Upregulated candidate genes with a similar expression pattern during plant stress responses. Several genes that match the expression profile are listed next to the graph. **(B)** Downregulated candidate genes with a similar expression pattern during plant stress responses. Several genes that match the expression profile are listed next to the graph.

To identify possible stress response candidate regulatory genes in kiwi, we performed a search for genes with expression patterns highly correlated with the plant response to both types of stresses. As shown in **Figure 4A**, considering the number of DEGs identified in each condition, kiwi plants increase their response to drought stress constantly from 0 to 24 hrs. of treatment (from T0 to T2), while the response to salt stress increases considerably more than that observed in drought stress at 6 hrs. of treatment (T1), but then decreases, returning to almost normal expression levels in T2 (**Figure 4A**).

Of the total DEGs identified, 142 candidate genes positively correlated with the plant response to both stresses. Within this group of genes, several ethylene-responsive transcription factors (ERF1B, ERF4, ERF10 and RAP2-1), NAC-domain containing proteins (NAP1, NAC002, NAC021, NAC025 and NAC083), WRKY DNA-binding proteins (WRKY7 and WRKY45), and A20/AN1 domain containing stress-associated proteins (SAP1 and SAP4.1) stood out (**Figure 4A**). On the other hand, the other 176 candidate genes that are downregulated present a similar pattern during the stress response (**Figure 4B**). This group includes basic helix-loop-helix DNA-binding proteins (bHLH19 and bHLH25), one MYB transcription factor MYB16, two TCP14transcription factors, and two candidate genes related to ABA (NCED4) and cytokinin (CKX7) regulation.

### 3.3 Identification of stress associated proteins in kiwi Hayward and expression under drought and salinity stress

**Figure 5:**
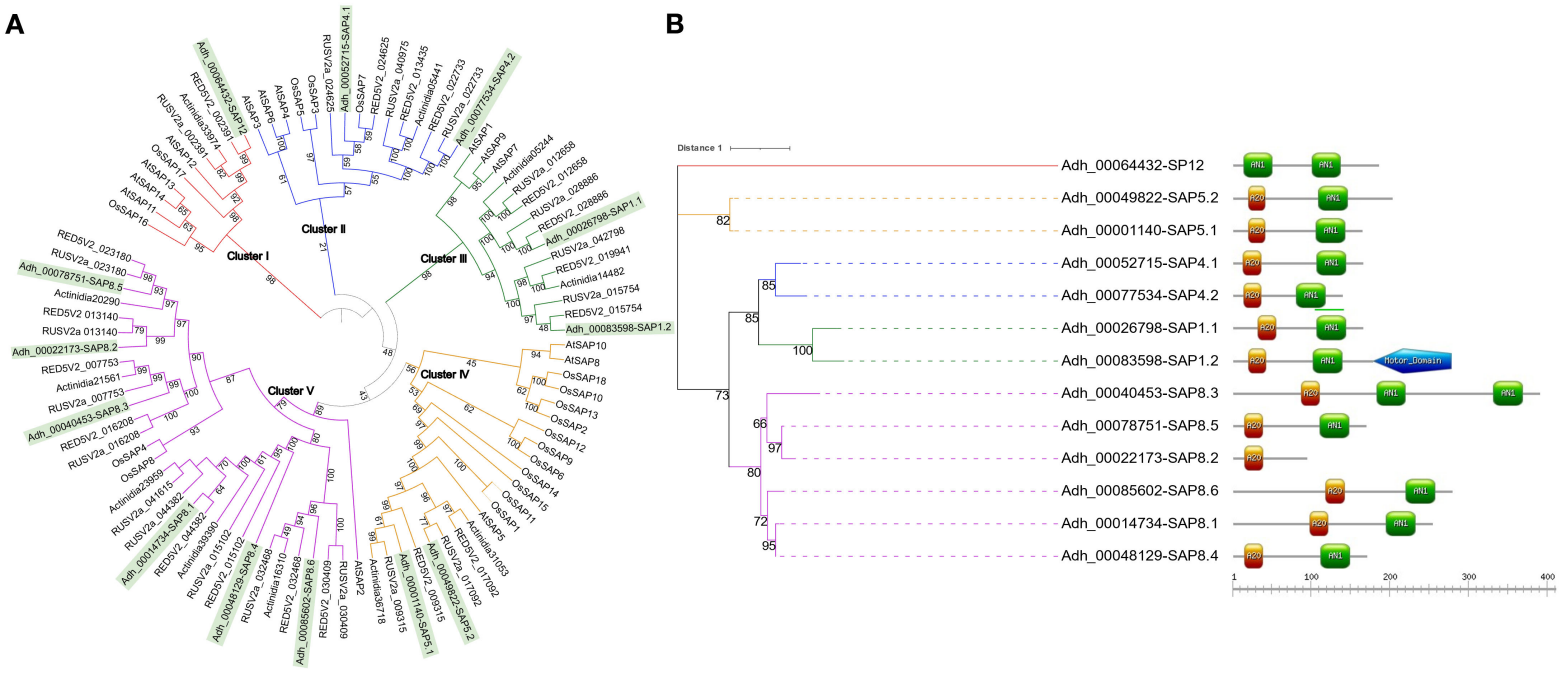
Phylogenetic relationships among SAPs family members. **(A)** Phylogenetic tree constructed using all SAPs detected from the annotation of *A*. *deliciosa* var. Hayward assembly, and *Oryza sativa* and *Arabidopsis thaliana* genomes. The analysis includes a total of 93 proteins. Bootstrap values over 51% are shown. Genes clustered into the 5 major clades where *Actinidia* SAPs are highlighted in green. **(B)** Phylogenetic representation of SAP members in *A*. *deliciosa* var. Hayward. The analysis includes a total of 13 proteins. Bootstrap values over 66% are shown. The clustering of genes is shown, employing the colors used in **Figure 2** for each protein cluster. On the right of each annotation number, the domains of each of the SAP proteins are depicted: the green rectangles represent the AN1 domains, the orange rectangles the A20 domains and the blue pentagon represents a predicted motor protein domain. Each member of the AdhSAP protein family was assigned a number based on their evolutionary relationships with the whole database of rice and Arabidopsis SAP proteins and a sub number counting the members of each clade (Sonnhammer and Koonin, 2002).

SAPs play a significant role in diverse plant stress responses and an increasing number of studies point to their association with tolerance or susceptibility to both abiotic and biotic stress. Therefore, we identified every SAP member of the Hayward variety in our genome assembly revealing a similar distribution found in other species where this family possesses in general 10 to 20 members (**Figure 5A**) (Ben Saad et al., 2024; Vij and Tyagi, 2006, 2008). To evaluate the evolutionary relationship of the AdhSAP family, a phylogenetic tree was constructed from the set of SAPs proteins, including those from *O. sativa* and *A. thaliana*. Sequences were distributed in five main clades, which are conserved when evaluating only the SAPs of A*. deliciosa* Hayward, thus identifying that the segregation of clades is associated with the difference in the distribution of their functional domains AN1 and A20 (**Figure 5B**). Interestingly, AdhSAP4.1 presents a high aminoacidic identity with OsiSAP7 (LOC_Os12g42400) (**Figure 5A**, Cluster II), one of the first known members of the SAP protein family that has been described to act as a negative regulator of abiotic stress responses (Geisler-Lee et al., 2007; Sharma et al., 2015). Considering the above, we selected *AdhSAP4.1* for further functional characterization.

### 3.4 Subcellular localization and overexpression of *AdhSAP4* in *N. tabacum*

**Figure 6:**
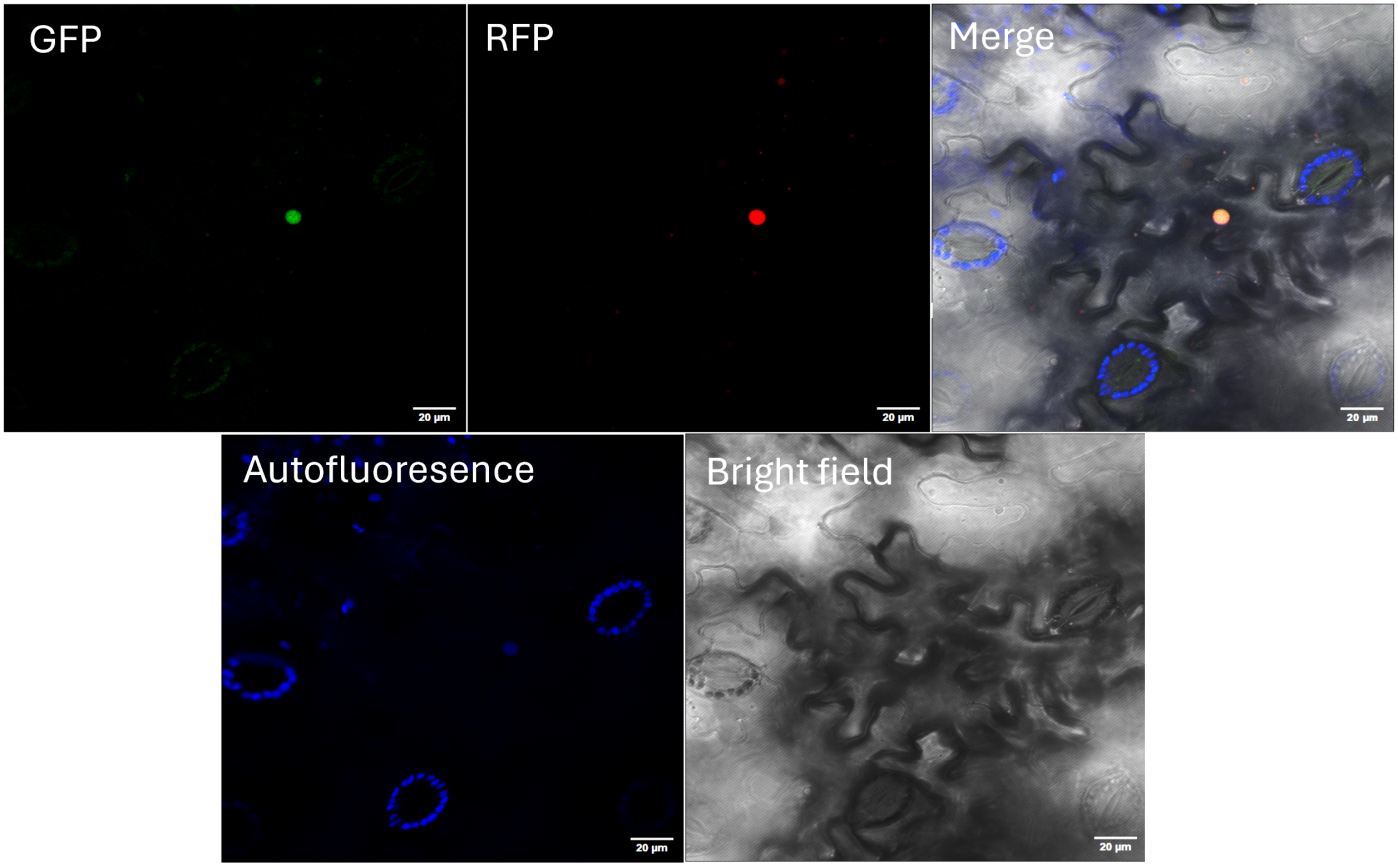
Subcellular localization of AdhSAP4-GFP fusion protein. Mature *Nicotiana benthamiana* leaves were co-infiltrated with Agrobacterium carrying the *p35S:AdhSAP4-GFP* (GFP) and *p35S:DcALFIN4-RFP* (RFP) vectors. Chlorophyll Autofluorescence and Bright field were also used to assemble the Merge image. Images were taken by confocal laser microscopy three days post-infiltration. *p35S:DcALFIN4-RFP* was included as a nuclear localization control (Quiroz-Iturra et al., 2022). Bars: 50 µm.

In most reports, SAP proteins are located in the nuclei despite the absence of nuclear transit signals in their aminoacidic sequence (Su et al., 2022). To determine the subcellular localization of AdhSAP4, transient expression of AdhSAP4-GFP in tobacco leaves was performed. As shown in **Figure 6**, AdhSAP4-GFP colocalized at the nucleus with DcAlfin4-RFP, a carrot transcription factor characterized by Quiroz-Iturra et al. (2022) suggesting that AdhSAP4 possesses nuclear localization.

**Figure 7:**
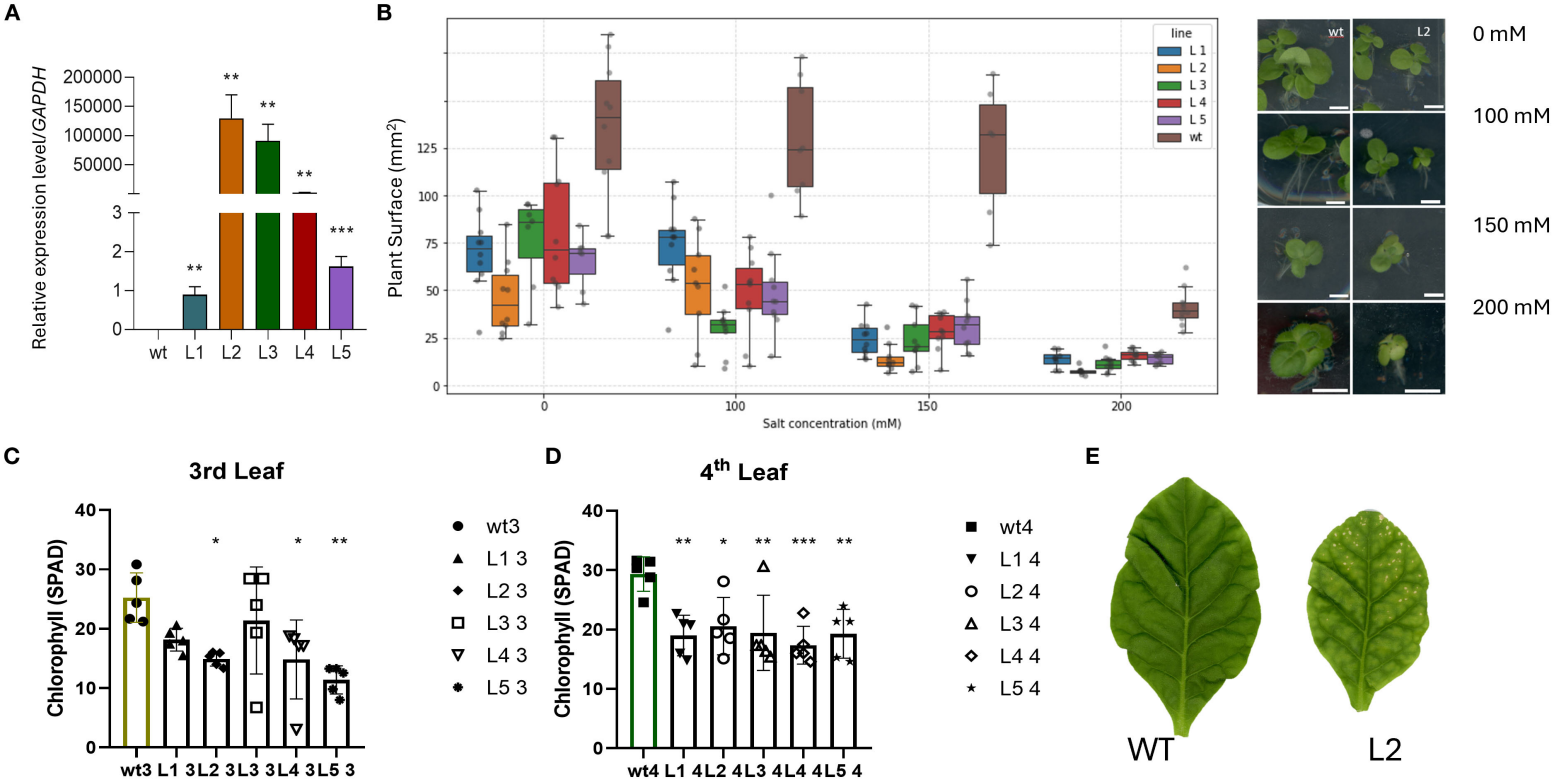
Relative expression of *AdhSAP4* and salt treatment in 35S::AdhSAP4 transgenic T1 tobacco lines. **(A)** Relative *AdhSAP4* transcript levels in each transgenic line (L1-L5). The expression assays were carried out in triplicate in a pool of 3–4 plants each and normalized with respect to the expression of *GAPDH*. Asterisks indicate statistically significant differences based on a student’s t-test between transgenic and WT plants (**p < 0.005, ***p < 0.001). For the chronic salt stress treatment, seedlings were grown on MS media for 10 days, then transferred to plates containing 0, 100, 150 and 200 mM NaCl and analyzed after three weeks. **(B)** Graphs represent the measurements of the plant area in mm^2^, and dark dots depict each plant (n = 10) after three weeks of treatment. The right panel shows a representative image of plant phenotype after treatment. A second chronic stress treatment was tested using one month old transgenic tobacco lines grown on MS media that were then transferred to rock wool pots and watered with hydroponic media for one month. After one month of growth, plants were then watered once every week with hydroponic media supplemented with 200 mM NaCl for two months until harvesting. The chlorophyll content was measured three times from the **(C)** third and **(D)** fourth leaves of 5 plants of each line using a SPAD Minolta monitor. **(E)** Representative image of the third leaves of WT and L2 plants after treatment. * p < 0.05.

To evaluate the effect of *AdhSAP4* as a positive or negative regulator of stress responses, we stably overexpressed *AdhSAP4* in *N. tabacum*. T0 transgenic tobacco plants harboring the *35S:AdhSAP4-GFP* construct were generated and selected (**Supplementary Figure S4**). Then, independent transgenic lines were cultivated until T1 seedlings were obtained which were used for functional and molecular assays. Initially, the expression of the transgene was evaluated in five T1 lines (**Figure 7A**), showing that L1 and L5 present a lower relative expression level of *AdhSAP4* than L2, L3, and L4 with respect to WT plants. Subsequently, we evaluated the susceptibility to salinity in overexpressing *AdhSAP4* lines. Twelve-day-old T1 seedlings from overexpressing lines grown on MS agar plates were transferred to MS plates supplemented with 0, 100, 150, and 200 mM NaCl for three weeks and monitored once per week (**Supplementary Figure S6**). By the third week, WT tobacco plants exhibited similar leaf areas (as an indication of growth) in 0, 100, and 150 mM NaCl; however, a significant reduction in growth was observed in the 200 mM NaCl treatment (**Figure 7B**). Regarding the transgenic lines, all present a reduced leaf area even at 0 mM NaCl when compared with WT, which could be associated with the metabolic effects generated by the overexpression of *AdhSAP4*. Under 100 mM NaCl, the leaf area in T1 and WT plants was similar than the control under 0 mM NaCl, with only L3 presenting a significant reduction in leaf area between 0 and 100 mM NaCl (**Figure 7B**). However, in the 150- and 200-mM treatments, a severe decrease in growth and the appearance of chlorosis in the leaves were evident and confirmed with a one-way ANOVA test and Tukey’s HSD analysis comparing the effect of the treatment on each individual line (**Supplementary Table S8**). Transgenic T1 lines not only present significant differences with respect to WT in each salt stress condition but also show a dramatic reduction in leaf area from 30–75 mm² at 100 mM to less than 30 mm² or 20 mm² at 150 mM or 200 mM of NaCl, respectively (**Figure 7B**). This effect, also accompanied by a similar reduction of root development (**Supplementary Figure S7**), seems independent of the transgene expression level suggesting that AdhSAP4 negatively impacts the plant stress response under salinity stress.

### 3.5 Long term exposure to salinity reduced the chlorophyll content in AdhSAP4 transgenic tobacco lines

Salinity produces several defects on plant growth and metabolism. Exposure to salinity is known to trigger a burst of reactive oxygen species (ROS) that can cause chlorophyll degradation (Verma and Mishra, 2005). Therefore, the chlorophyll content was determined in two-month-old AdhSAP4 lines that had been irrigated with 200 mM of NaCl twice a week for four weeks. By the end of the 4^th^ week of treatment, we measured chlorophyll in the third and fourth leaves (counted from bottom to top) which averaged 6 leaves per plant. We selected two types of leaves for this analysis since stress often causes symptoms that vary with leaf position between younger and older leaves (Jansen et al., 2009). The third leaves of the AdhSAP4 lines were evidently stressed with a reduced leaf size and chlorosis symptoms (**Figure 7B** right panel). Especially, the chlorophyll content of the third leaves was decreased significantly (**Figure 7C**), and even more so in the fourth leaves (**Figures 7D, E**). This leads us to conclude that AdSAP4 is a negative regulator conferring susceptibility to salt stress and decreases chlorophyll levels.

## 4 Discussion

### 4.1 *A. deliciosa* var. Hayward genome assembly

Most of the sequenced genomes of the genus *Actinidia* belong to the species *chinensis*, including the recent genome of *A. chinensis* var. ‘Chinensis’ (Xia et al., 2023) as well as the previously sequenced varieties like ‘Russel’, ‘Red5’ and ‘HongYang’. The present work presents a haploid genome assembly of *A. deliciosa* var. Hayward. This variety was selected by Hayward Wright, a nurseryman from Auckland (NZ) from early cultivated female plants, probably pollinated by a single male. Almost all the *A. deliciosa* cultivars cultivated outside China are the descendants of these plants introduced in New Zealand in the early 1900’s (Ferguson, 1999). This distinctive origin is evidenced by comparing the orthologous genes of the respective genomes, showing a more ancient evolutionary divergence than the members of the *A. chinensis* species (**Figure 1**).

Polyploidy plays an important role in the evolution of plant genomes, increasing the adaptive plasticity to extreme environments and the summative effect of its duplicated genes (Zhang et al., 2019; Nieto Feliner et al., 2020; Heslop-Harrison et al., 2023). Polyploidy can also cause massive rearrangements in transcriptomic and regulatory mechanisms, likely triggered by imbalanced amounts of regulatory elements like transcription factors, small RNAs and others, modifying from single-gene expression to entire networks of altered regulatory modules (Nieto Feliner et al., 2020). This genomic coalescence implies that the assembly and annotation of a polyploid organism is not a trivial problem, leading to the search for specific methods to determine the evolutionary origin of its genetic duplications (Leal et al., 2024). Even so, much of the initial effort in the genetic description of a polyploid species is usually to obtain an initial haploid genome (Kyriakidou et al., 2018).

A recent study showed that a significant portion of the *A. deliciosa* hexaploidy genome aligns with the diploid *A. chinensis*, with 95.5% of homologous gene pairs showing over 90% similarity. However, comparisons within and between genomes reveal chromosomal modifications suggesting that if *A. deliciosa* is a probable autoploid, chromosomal rearrangements happened post-autohexaploidy (Liu et al., 2024). At the annotation level, the haploid genome of *A. deliciosa* var. Hayward has a high completeness (96.2% BUSCO), with a number of annotated proteins similar to those of other *Actinidia*, from which it diverged more than 10 MYA ago, similar to that described in *A. chinensis* var chinensis with respect to the Red5 and HongYang cultivars (Liu et al., 2024; Xia et al., 2023).

Regarding the Stress Associated Proteins, we observed that the orthologous groups of SAPs in kiwi Hayward do not show expansion or contraction of their gene families. This is likely due to the genetic closeness of the species and the fact that the current assembly is based on a haploid version of the genome, which may miss some of the actual copy diversity resulting from the whole genome duplication that led to their allohexaploidy. Considering the presence at the N or C terminus of the A20 and AN1 domains, a total of 13 kiwi *AdhSAPs* can be divided into 5 clusters (groups I to V) in phylogenetic relationships (**Figure 5**) (Su et al., 2024). The A20 domain contains seven characteristic Cys2/Cys2 zinc fingers at its C-terminal with a conserved CX_2-4_CX_11_CX_2_C domain. On the other hand, the AN1 domain was first discovered as a ubiquitin-like fusion protein of *Xenopus laevis* eggs and early embryos (Linnen et al., 1993). Although the roles of some of these A20/AN1 zinc-finger proteins in animal systems have been related to the regulation of immune responses, their function in plants has not yet been completely clarified (Vij and Tyagi, 2008).

Each of the SAPs identified in the genome of *A. deliciosa* Hayward is phylogenetically associated with SAPs of *A. thaliana* or *O. sativa*, which allows us to classify their possible functionality, highlighting Cluster V as the most diverse in protein domains, where all *A. deliciosa* Hayward SAPs in this cluster were identified as homologs to OsSAP4 and OsSAP8 (**Figure 5**). The OsiSAP7 gene of the *O. sativa* has been characterized as a negative regulator of abiotic stress, where its overexpression generates susceptibility to salt stress (Ben Saad et al., 2024; Li et al., 2022; Sharma et al., 2015). The closest homolog of OsiSAP7 in *A. deliciosa* var. Hayward is the gene Adh_00052715, identified as *AdHSAP4*, both found in the clade of Cluster II (**Figure 5**). This evidence supported our interest in characterizing the role of *AdHSAP4* in salt stress.

### 4.2 Differential expression analysis of *A. deliciosa* var. Hayward under salinity and drought

Differential expression analysis allowed us to identify a total of 318 candidate genes (CG) (**Figure 4**) with expression patterns highly correlated with the development of the response to both saline and drought stresses. An overrepresentation of genes associated with the response to ROS was identified (as observed in the GO analysis of **Figure 3** for both stresses). Redox homeostasis reduces ROS levels, generally through antioxidant enzymes like superoxide dismutases (SOD), peroxidases (POD), catalases (CAT), ascorbate peroxidases (APX), peroxiredoxins (PRX), glutathione peroxidases (GPX), and glutathione S-transferases (GST). These enzymes actively remove excess ROS to protect plant cells from oxidative damage (Hasanuzzaman et al., 2020). In this sense, within the 318 CG identified, 142 were positively correlated with both stresses and measurement times, along with four cytochrome P450 family proteins (AcDH_00048223, AcDH_00014316, AcDH_00084161 and AcDH_00032701) and one gene described as a raffinose synthase family protein DIN10 (AcDH_00046123). Cytochrome enzymes play diverse roles, including the metabolism of hormones, fatty acids, sterols, cell-wall component biopolymers, and defense-related compounds such as terpenoids and flavonoids. In *Glycine max*, a P450 family member has been shown to participate in abiotic stress responses in the Jasmonic Acid and Ethylene signaling pathway (Yan et al., 2016) and in sweet orange, a member of this family participates in the metabolism of antioxidant flavonoids and contributes to drought tolerance by augmenting ROS scavenging activities (Pandian et al., 2020; Rao et al., 2020). On the other hand, DIN10 is involved in the synthesis of raffinose, associated with osmotic adjustment to avoid oxidative damage caused by ROS via the synthesis of raffinose as osmolyte (Nishizawa et al., 2008; Li et al., 2020). All these genes highlight the oxidative scenario observed under both stress treatments.

On the other hand, polyubiquitin genes are known to participate in the turnover of proteins by the ubiquitin proteasome system (UPS) (Ponnu and Hoecker, 2021; Wang et al., 2024). This is the same as stress associated proteins like SAP5 (AcDH_00049822) which are present in the most expressed tier in drought stress treatments, and are known to participate in drought and salinity stress tolerance as a positive regulator of stress with E3 ubiquitin ligase activity in *Arabidopsis* (Yang et al., 2023).

### 4.3 AdhSAP4 confers susceptibility to salt stress in *N. tabacum*

A20/AN1 proteins are part of complex regulatory networks since they can be activated by phosphorylation by receptor-like cytoplasmic kinases (Giri et al., 2013) and plants rely heavily on regulatory mechanisms such as the UPS to maintain cellular homeostasis and continual growth under adverse conditions. The UPS is used to regulate the function of proteins involved in generating the cellular changes required to respond to the changing environment and mitigate the negative impact of stress.

Recent studies have demonstrated that SAPs contribute toward several cellular processes like tolerance to drought, osmotic stress (Dong et al., 2018; Su et al., 2022), water retention (Lloret et al., 2017), salt tolerance (Li et al., 2019), and temperature regulation (Bae et al., 2021). Their functional diversity is intriguing, particularly given their roles as ubiquitin ligases, redox sensors, and gene expression regulators (Kang et al., 2017; Bae et al., 2021). Some SAPs can also translocate into the nucleus and bind to stress-responsive genes, adding an additional layer of complexity to their roles. This range of cellular functions makes it challenging to pinpoint the exact roles of many SAPs. The specific protein partners with which SAP proteins interact remain unknown, as does the possibility of interactions between SAP proteins themselves (Giri et al., 2013). Additionally, some SAP proteins have been observed to translocate into the nucleus and bind to cis-acting elements of stress-responsive genes (Vij and Tyagi, 2008), but their exact role in gene expression is still unknown. Taking this information into account, we selected AdhSAP4 to decipher its potential role as a positive or negative regulator to salinity in plants. *N. tabacum AdhSAP4* overexpression lines displayed sensitivity to salt stress and a reduced chlorophyll content (**Figure 7**). This negative effect has only been described in a few A20/AN1 proteins like in OsiSAP7, the closest homolog of AdHSAP4. Specifically, Sharma et al. (2015) evaluated *OsiSAP7* and found evidence to support OsiSAP7 as a negative regulator of ABA and water deficit stress signaling with E3 ligase activity. Overexpression of *OsiSAP7* has been linked to a negative regulatory role that manifests as reduced chlorophyll content in overexpressing plants (Kang et al., 2017; Li et al., 2019). Mechanistically, *OsiSAP7* overexpression increases the hypersensitivity of plants to reactive oxygen species, a condition that predisposes the chloroplast to oxidative damage and subsequent degradation of chlorophyll molecules. This ROS hypersensitivity is attributed to an imbalance in the ROS detoxification mechanisms; for instance, unlike positive SAP regulators such as *OsSAP1* and *OsSAP8* that upregulate antioxidant enzymes like catalase (CAT), superoxide dismutase (SOD) and peroxidases (POD) (Muthuramalingam et al., 2021; Chen et al., 2021), *OsiSAP7* overexpression may fail to adequately induce such protective responses, thereby allowing ROS levels to rise unchecked. Consequently, the oxidative damage incurred under these conditions leads to a more rapid degradation of chlorophyll, compromising the photosynthetic machinery. Similarly, *Prunus* PpSAP1 and *O. sativa* ZPF185 when overexpressed produced a smaller leaf size and increased sensitivity to abiotic stress, similar to our observations when overexpressing AdhSAP4 in tobacco (**Figure 7**, **Supplementary Figures S6, S7**) supporting the dual roles of SAP proteins in stress and developmental processes which are unexpectedly diverse. Therefore, our results suggest that *AdHSAP4* is a negative regulator as well, which could be further studied as a candidate for *A. deliciosa* crop improvement through gene editing.

## 5 Conclusions

In this study the assembly and annotation of a high-quality *A. deliciosa* var. Hayward was obtained by integrating PacBio and Illumina sequencing leading to an improvement in the number of genes and coverage in contrast with previous *A. chinensis* genome versions. The RNA sequencing of kiwi leaves exposed to high salinity and drought conditions revealed a glimpse into the responses of kiwi to manage the stress conditions with different sets of responses in each case. From the Gene Ontology analysis, phytohormones showed a similar response to both drought and salinity stress treatments, highlighting their crucial regulatory role in kiwi Hayward. Jasmonic acid, organic hydroxy compounds, toxin metabolic processes, and responses to salicylic acid, ethylene, fatty acids, and cold are significantly overrepresented in both stress conditions, suggesting that these pathways are critical for stress responses. The sum of observations from the transcriptomic analyses suggests that while both types of stress elicit similar initial responses involving phytohormones and the adjustment of metabolic processes, the timing and duration of specific responses such as hypoxia and photosynthesis vary between drought and salinity stress in kiwi (Mustroph et al., 2009; Wurms et al., 2023). This indicates a complex and dynamic adaptation mechanism in Hayward plants to manage different environmental stresses.

Finally, the A20/AN1 protein AdhSAP4 is localized in the cell nucleus although it lacks canonical nuclear transit signals. Moreover, the over expression of *AdhSAP4* increased sensitivity to salt stress, impaired leaf size and reduced chlorophyll content in *N. tabacum* highlighting *AdhSAP4* as a negative regulator of salt stress tolerance in *A. deliciosa* var. Hayward.

## Data Availability

The datasets presented in this study can be found in online repositories. The names of the repository/repositories and accession number(s) can be found below: https://www.ncbi.nlm.nih.gov/genbank/, PRJNA1244473.
