# Supplementary material for: Decoding the Hayward kiwi (Actinidia deliciosa var Hayward) genome: transcriptomic responses to drought and salinity and AdhSAP4’s role in salinity stress responses
Source: Front Plant Sci. 2025 Sep 23;16:1637092. doi: 10.3389/fpls.2025.1637092 (PMC12500665; doi:10.3389/fpls.2025.1637092)
Supplement: Supplementary file 1 [file DataSheet1.zip › Supplementary Figures.docx]

Supplementary figures


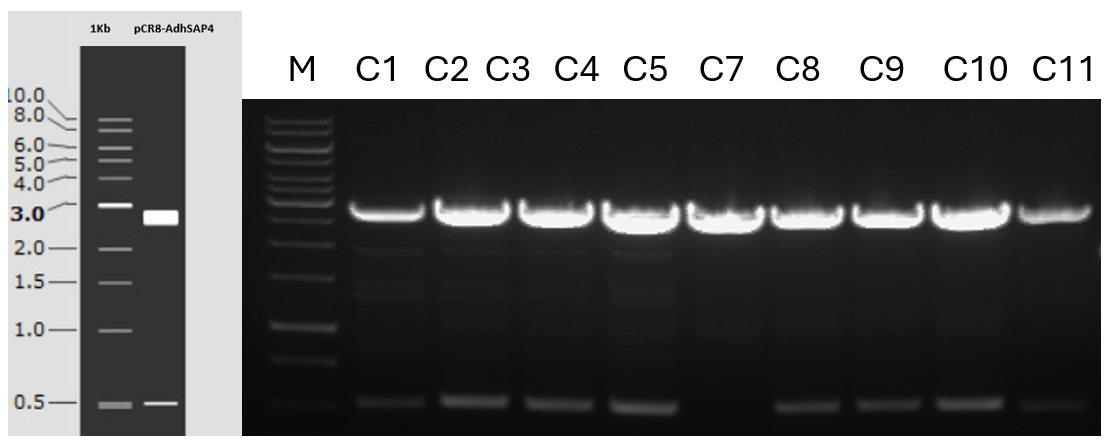


**Supplementary Figure** **1: Enzymatic digestion of plasmid DNA extracted from pCR8-AdhSAP4 *E. coli.* colonies.** On the left is the expected digestion pattern obtained using the Snapgene software (GSL Biotech) if the colonies have the insert. On the right is the digestion of *E. coli* colonies 1-11 with EcoRI. Arrows indicate sizes of 3000 bp and 500 bp. 1 kb: GeneRuler 1 Kb DNA Ladder molecular weight marker. C11 was used afterwards.


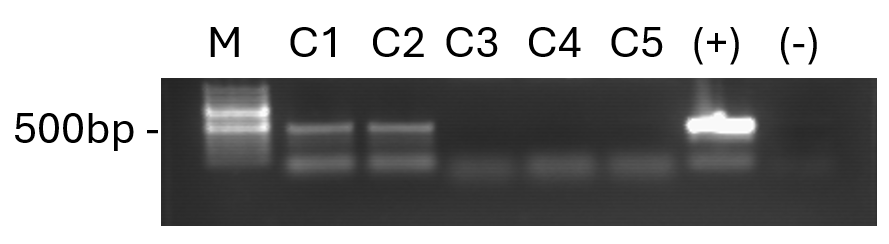


**Supplementary Figure 2**: **Amplification of *A. tumefaciens* colonies transformed with AdhSAP4:GFP vector.** C1-C5: colonies analyzed by amplification of the 500bp *AdhSAP4* gene. C+: positive reaction control (pCR8-AdhSAP4 vector), C-: negative reaction control (reaction without DNA, only water). MP: GeneRuler 100 bp DNA Ladder molecular weight marker.


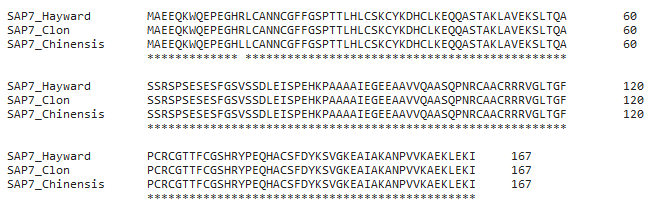


**Supplementary Figure 3:** **Alignment of the predicted AdhSAP4 protein sequence**. An alignment of the predicted protein sequence obtained from the genome annotation (Hayward), a sequenced pCR8 cloned sequence (Clon) and the sequence of the *Actinidia chinensis* Red5 annotated genome (Chinensis) obtained using the Geneious prime alignment tool with standard Global alignment with free gaps with a penalty of 15 and extension penalty of 1 for gaps using the Blosum62 cost matrix.


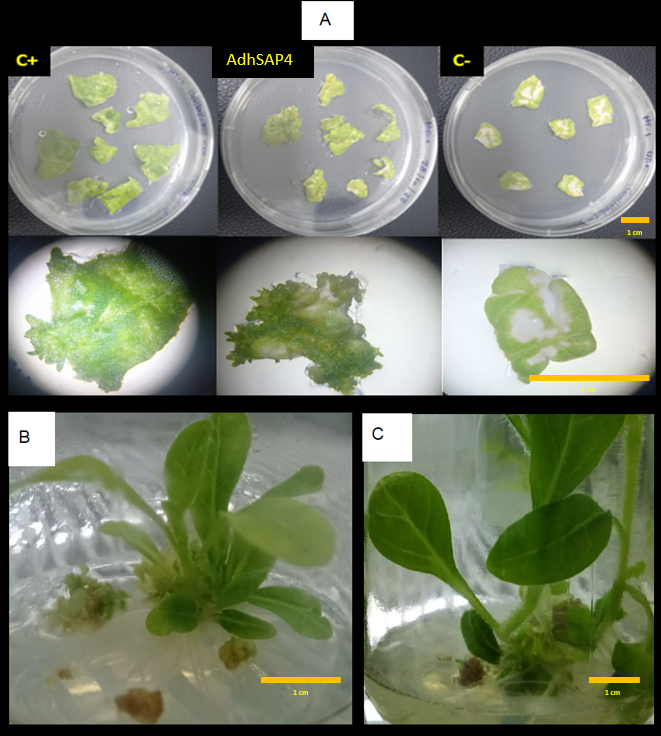


**Supplementary Figure 4.** **Transformation and regeneration of 35S:AdhSAP4 tobacco lines**

A) Explants of 1 month old tobacco plants were transformed using the pGWB5-AdhSAP4 vector and an empty version of the pGWB5 vector as a negative control (C-). C+: Regeneration of plants from non-transformed explants. B) and C) Examples of T0 regenerated transformed plants before they were adapted to soil conditions.


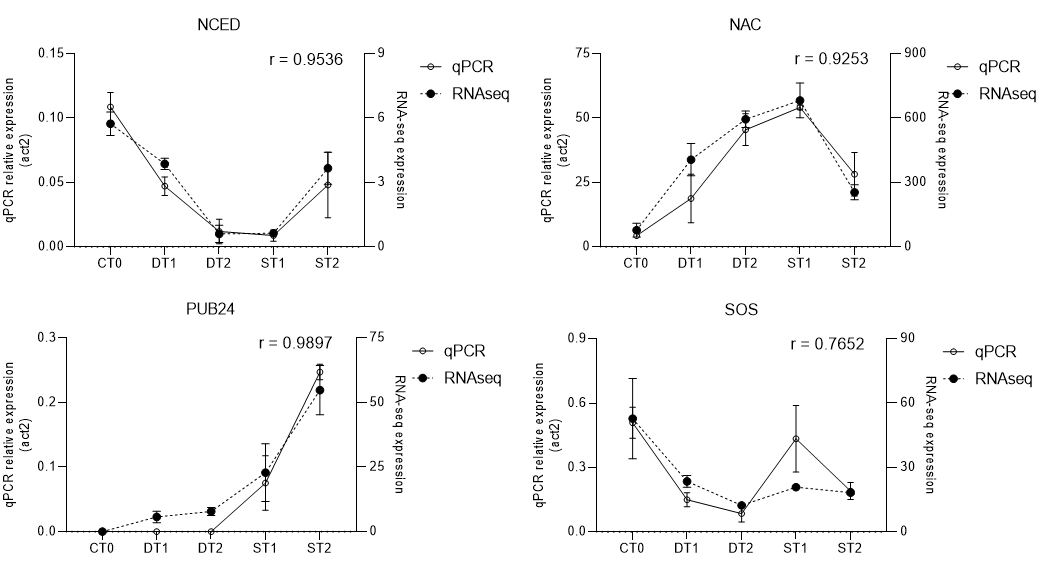


**Supplementary Figure 5. Differentially expressed genes in kiwi Hayward transcriptome validated by qRT-PCR.** Four candidate genes obtained from transcriptomic analysis were used to validate RNA-seq expression data (dashed lines/closed circles) with qRT-PCR expression (solid lines(open circles). Pearson correlations were used to validate expression patterns obtained with both analyses and are represented with an r-value located in the upper right section of each graph. PUB24, (AcDH_00061942); NCED, (AcDH_00020801);  NAC, (AcDH_00020394); SOS, (AcDH_00000990a)


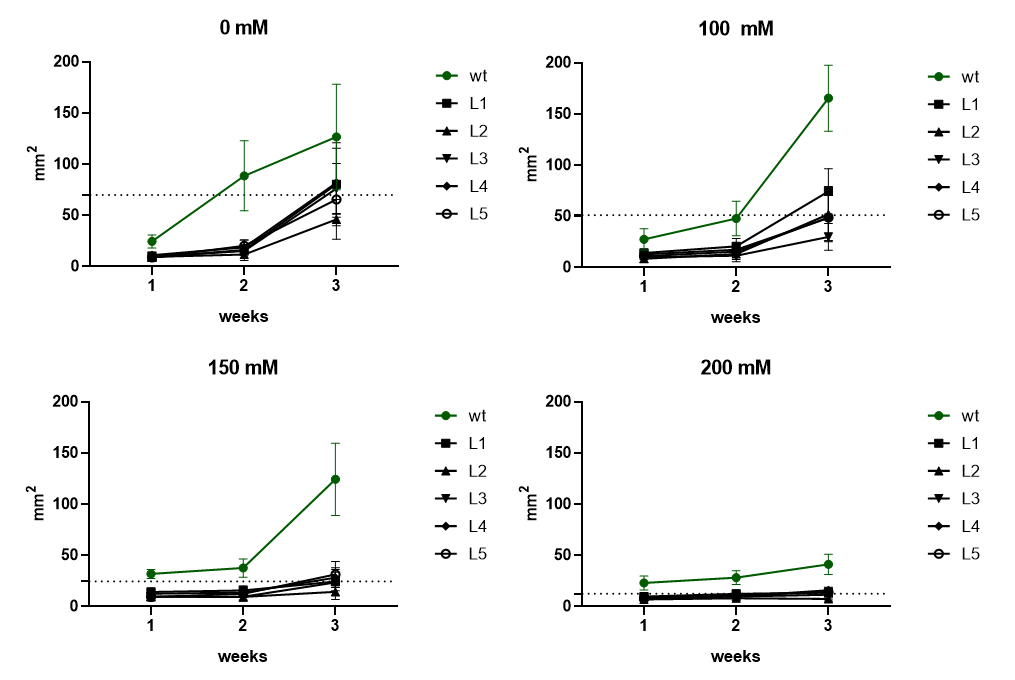


**Supplementary Figure 6**. **Effect of salinity on the leaves area of** **tobacco AdhSAP4 transgenic lines.** Transgenic T1 tobacco AdhSAP4 transgenic lines (L1-L5) were grown on MS media for 10 days, then transferred to plates containing MS supplemented with 0, 100, 150 or 200 mM NaCl. Total leaf area was measured every week for three weeks. Each point represents 10 plants. The dotted line represents the mean growth area of all AdhSAP4 transgenic lines by the third week (L1-L5 ).


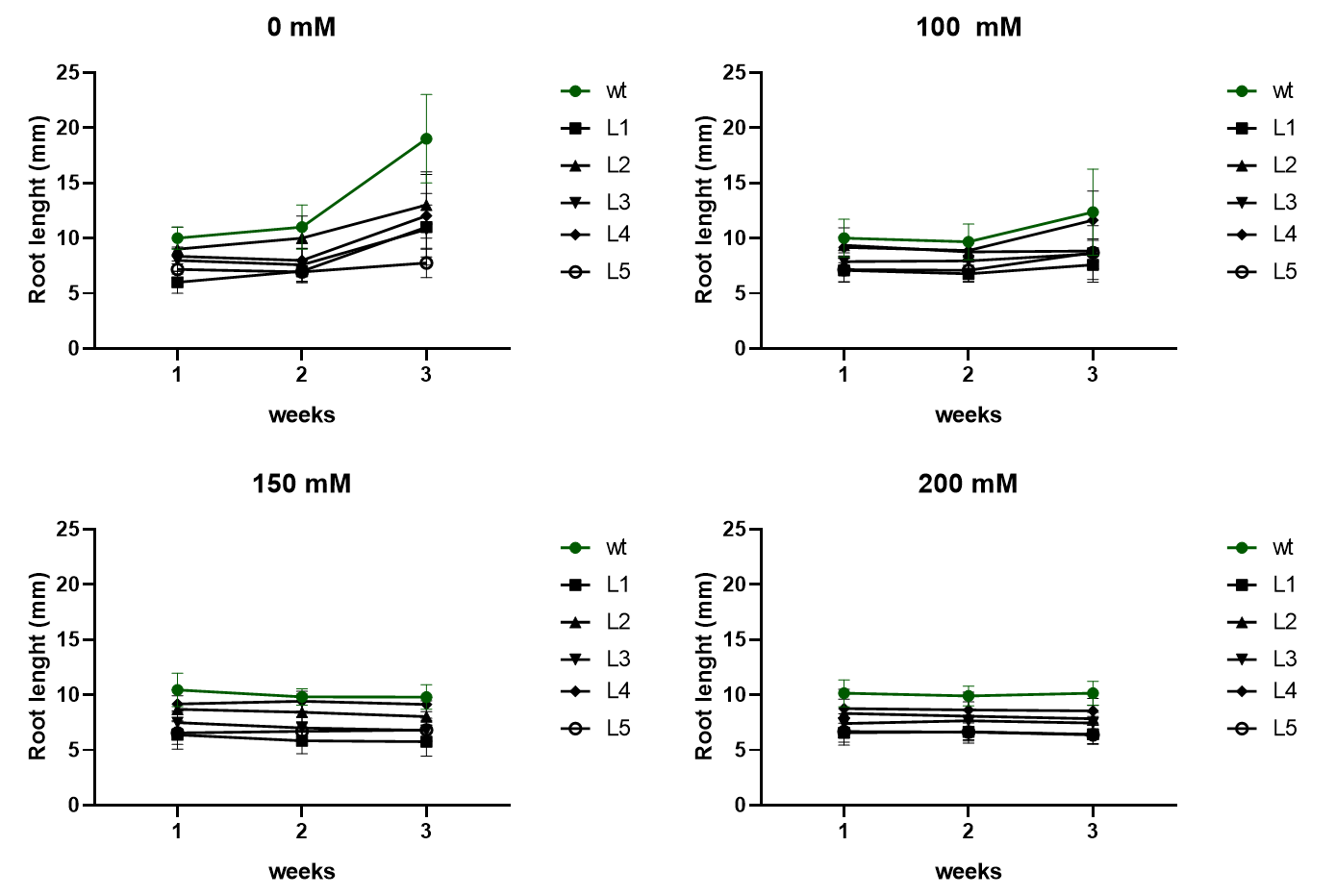
**Supplementary Figure 7**. **Effect of salinity on the root growth of** **tobacco AdhSAP4 transgenic lines.** Transgenic T1 tobacco AdhSAP4 transgenic lines (L1-L5) were grown on MS media for 10 days, then transferred to plates containing MS supplemented with 0, 100, 150 or 200 mM NaCl. Root length was measured every week for three weeks. Each point represents 10 plants.


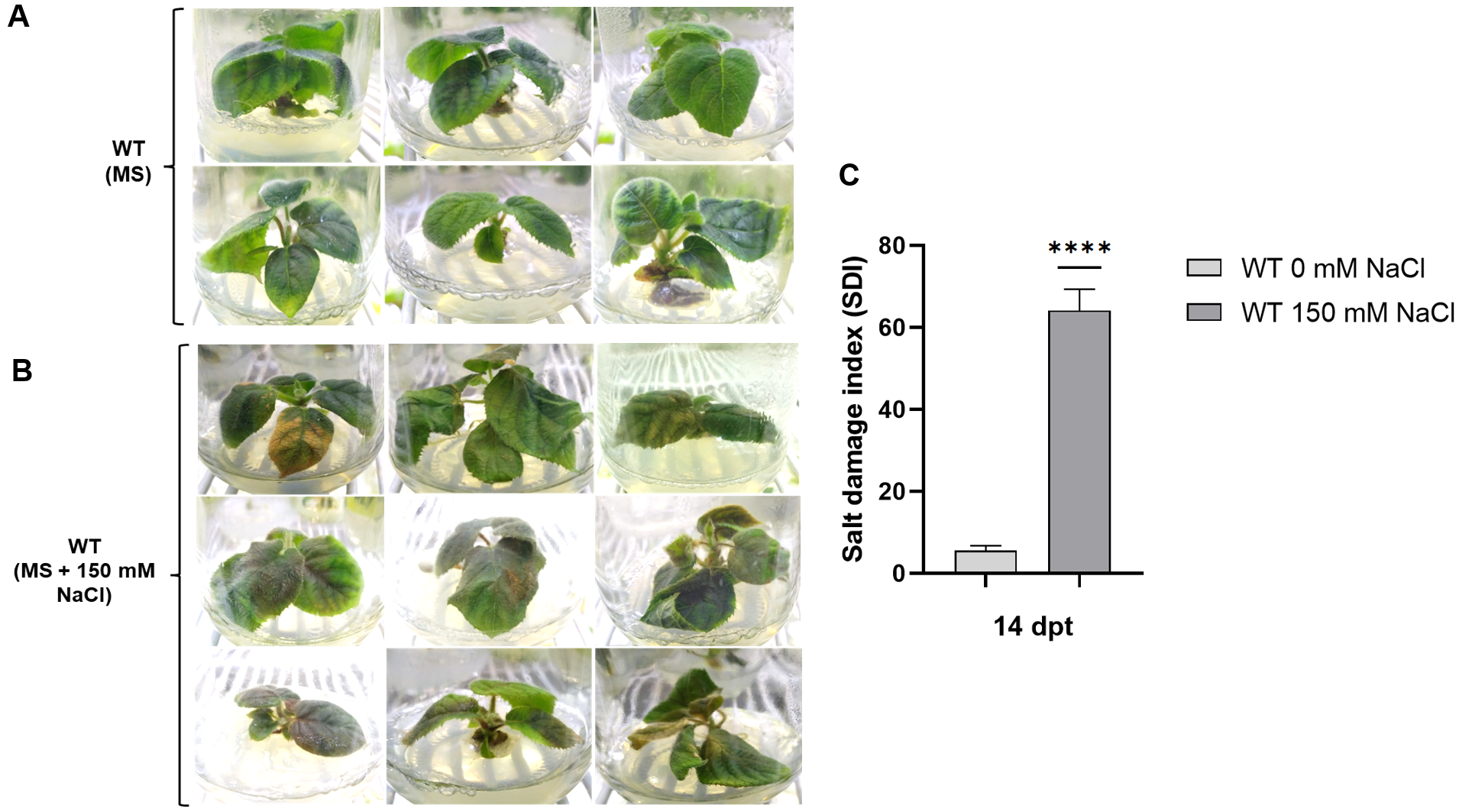


**Supplementary figure 8. Salinity Tolerance Assay in In vitro grown Hayward kiwi plants (*Actinidia deliciosa*).**Photographs of Hayward kiwi plants (*Actinidia deliciosa*) cultivated in MS medium **(A)** and MS medium supplemented with 150 mM NaCl **(B)** for 14 days under in vitro conditions. Damage index quantification under salinity stress **(C)** at 14 days post-treatment, based on the method described by Abid *et al*. (2020). Data are presented as mean ± standard deviation for *n* = 18 leaves (6 plants) in the control group and *n* = 27 leaves (9 plants) in the NaCl-treated group. Statistical differences were evaluated using a *t*-test, with **** indicating *p* < 0.0001.
